# Role of proline in cell wall synthesis and plant development and its implications in plant ontogeny

**DOI:** 10.3389/fpls.2015.00544

**Published:** 2015-07-20

**Authors:** Polavarapu B. Kavi Kishor, P. Hima Kumari, M. S. L. Sunita, Nese Sreenivasulu

**Affiliations:** ^1^Department of Genetics, Osmania University, HyderabadIndia; ^2^Leibniz Institute of Plant Genetics and Crop Plant Research, GaterslebenGermany; ^3^Grain Quality and Nutrition Center, International Rice Research Institute, Metro ManilaPhilippines

**Keywords:** proline, plant ontogeny, proline-rich proteins, hydroxylproline-rich glycoproteins, hybrid proline-rich proteins

## Abstract

Proline is a proteogenic amino acid and accumulates both under stress and non-stress conditions as a beneficial solute in plants. Recent discoveries point out that proline plays an important role in plant growth and differentiation across life cycle. It is a key determinant of many cell wall proteins that plays important roles in plant development. The role of extensins, arabinogalactan proteins and hydroxyproline- and proline-rich proteins as important components of cell wall proteins that play pivotal roles in cell wall signal transduction cascades, plant development and stress tolerance is discussed in this review. Molecular insights are also provided here into the plausible roles of proline transporters modulating key events in plant development. In addition, the roles of proline during seed developmental transitions including storage protein synthesis are discussed.

## Introduction

Glutamate is an important amino acid and acts as a precursor for the biosynthesis of γ-aminobutyric acid, arginine, glutamine, and proline in plants as well as in other eukaryotes (Rhodes et al., 1986; Szekely et al., 2008; Sharma and Verslues, 2010). Proline can be distinguished among all other amino acids due to its unique structure with its α-amino group as a secondary amine and possesses distinctive cyclic structure which causes exceptional conformational rigidity to the protein structure (MacArthur and Thornton, 1991). It has also specific chemical properties like zwitterionic nature, neutral pH, high compatibility with cell milieu and extreme solubility as 14 kgs of it can be dissolved in 1 kg of water (LeRudulier et al., 1984). Though evidence has been presented for the synthesis of proline from glutamate and ornithine in higher plants (Delauney and Verma, 1993; Sharma et al., 2013), the role of ornithine as proline precursor is doubtful as pointed out by Funck et al. (2008). The pathway for proline biosynthesis in higher plants differs from that of bacteria (Kishor et al., 2005; Verbruggen and Hermans, 2008; Szabados and Savoure, 2010). The genes that encode the enzymes involved in proline biosynthesis like pyrroline-5-carboxylate synthetase (*P5CS*), pyrroline-5-carboxylate reductase (*P5CR*), and ornithine-δ-aminotransferase (*OAT*) and proline catabolism such as proline dehydrogenase (*PDH*) and pyrroline-5-carboxylate dehydrogenase (*P5CDH*) have been identified and isolated (Delauney and Verma, 1990; Hu et al., 1992; Delauney et al., 1993; Kiyosue et al., 1996; Strizhov et al., 1997; Deuschle et al., 2001; Funck et al., 2010). *P5CS1* was first cloned from *Vigna aconitifolia* by complementation technique (Hu et al., 1992), and found to be a novel bifunctional enzyme (with activities of both γ-glutamy kinase and glutamic γ-semialdehyde dehydrogenase) that catalyzes glutamate to pyrroline-5 carboxylate, an intermediate in proline biosynthesis. This is a rate limiting step in proline biosynthesis. P5CS is encoded by two differentially regulated genes (*P5CS1* and *P5CS2*) and *P5CS2* was first cloned from *Arabidopsis thaliana* (Strizhov et al., 1997). While *P5CS1* is abundantly expressed in most plant parts but not in dividing cells, *P5CS2* is highly expressed at the transcriptional level in dividing cells (Strizhov et al., 1997). Besides acting as a proteogenic amino acid, it accumulates in large quantities and plays a role during abiotic stress tolerance in plants (Kishor et al., 1995, 2005; Sharma and Verslues, 2010). Thus, it is known that proline participates in the biosynthesis of primary metabolism, but also has special functions to carry as a metabolite during growth and development (Hare and Cress, 1997; Hare et al., 1999; Trovato et al., 2001; Mattioli et al., 2009a; Funck et al., 2012). Proline not only accumulates during abiotic stress, but also in different tissues of plants under non-stress conditions too (Kishor and Sreenivasulu, 2014). The role of proline as osmoprotectant in stress tolerance has been extensively reviewed (Verbruggen and Hermans, 2008; Szabados and Savoure, 2010). Though many amino acids constitute a major source of the components used for cellular growth and differentiation in higher plants, proline is distinct from others. The emerging roles of proline in plant growth and development from the perspective of proline homeostasis in mediating growth and development has been reviewed (Kishor and Sreenivasulu, 2014). Over and above, proline is an important source of cell wall matrix. As a component of cell wall proteins, it plays pivotal role in plant development but understanding of its diverse functions appears to be enigmatic. Cell walls contain hydroxyproline-rich *O*-glycoproteins (HRGPs) as complex macromolecules with varying structures and functions and encompass a broad category of extracellular proteins. HRGPs contain variations of Pro-Pro repeats (Cassab and Varner, 1988; Showalter, 1993) and are lightly glycosylated. Their classification is based on proline residue-containing proteins (Cassab and Varner, 1988; Showalter, 1993). It is a superfamily that is classified into extensins (EXTs) that are moderately glycosylated, arabinogalactan-proteins (AGPs) that are hyperglycosylated (Henrissat et al., 2001; Lamport et al., 2011) and Hyp/Pro-rich proteins (H/PRPs) that may not be glycosylated at all, or either weakly- or highly glycosylated. HRGPs undergo post-translational modifications like conversion of proline to hydroxyproline (Hyp) by a membrane-bound prolyl 4-hydroxylases (P4Hs) or glycosylation of HRGPs by glycosyltransferases (Rhee et al., 2003; Mayer and Jurgens, 2004; Wang and He, 2004). This sub-family is characterized by *O*-Hyp linked arabinosides and arabinogalactan polysaccharides (Lamport, 1967, 1977; Pope, 1977) which define the molecular surface. Arabinosylation and arabinogalactosylation would depend upon contiguous Hyp and clustered non-contiguous Hyp. What directs the Hyp glycosylation is a code based on peptide sequence. While Ser-Hyp_4_ motifs of EXTs are preferred sites for the addition of arabinoside, Ser-Hyp and Ala-Hyp repeats of the AGPs are the usual sites of arabinogalactan heteropolysaccharide addition (Kieliszewski and Shpak, 2001; Zhao et al., 2002; Held et al., 2004). Studies carried out by many suggest that species variation and tissue specific Hyp glycosylation occurs (Kieliszewski and Shpak, 2001; Zhao et al., 2002; Held et al., 2004). HRGPs appear to be not solubilized in cell walls as a response to the stress (Showalter, 1993; Fowler et al., 1999). They are expressed during wounding and pathogen attack in some tissues and implicated in different stages of plant growth and development, nodule formation, fertilization, cytokinesis, apoptosis, senescence, and cell wall lignification (Ye et al., 1991; Showalter, 1993, 2001; Nothnagel, 1997; Wu et al., 2001; Hall and Cannon, 2002; Lamport et al., 2006). Different roles of proline in plant ontogeny and during key transitions are summarized in **Table 1**. In the present endeavor, we aim at reviewing the progress of multidimensional functions of proline in plant ontogeny which is contributing to cell wall modifications and also during key transitional events especially reproductive development. In addition, the implications of proline during seed development and seed storage metabolism has been highlighted.

**Table 1:** Multidimensional roles of proline in different cell organelles, tissues, organs, and whole plants.

Cell organelle/tissue/organ	Proline/proline-rich/hydroxyproline (Hyp)-rich proteins	Function	Reference
Cell wall	PRPs	Wall components	McCann et al. (2001)
Cell	PRPs	Cell elongation	Ito et al. (1998)
Cell	PRPs	Root hair development	Bernhardt and Tierney (2000)
Whole plant	PRPs	Abscission and senescence	Merkouropoulos and Shirsat (2003)
Whole plant	PRPs	Development and abiotic stress tolerance	Zhan et al. (2012)
Floral buds	PRPs	Structural integrity of the style	Cheung et al. (1993)
Flower	PRPs	Flower development	Fowler et al. (1999)
Flower	PRPs	Flower development	Gothandam et al. (2010)
Flower	PRPs	Cotton fiber development	Xu et al. (2013)
Phloem	PRPs	Expression in phloem in response to drought stress	Battaglia et al. (2007)
Cell wall	PRPs	Drought stress	Creelman and Mullet (1991), Colmenero-Flores et al. (1997)
Plasma membrane	Repetitive proline-rich proteins (PRPs)	Sensitivity of roots to ABA	Tseng et al. (2013)
Plasma membrane	HRGPs	Plasma membrane-cytoskeleton continuum	Showalter (1993), Showalter et al. (2010)
Cell	HRGPs	Wall assembly and remodeling during cell growth	Doblin et al. (2010), Ellis et al. (2010)
Cell	HRGPs	Cell-cell integrations and communications	Wu et al. (2001)
Pollen and style	HRGPs	Pollen tube and style growth	Zhang et al. (2014)
Callus/suspensions	HRGPs	Somatic embryogenesis, germination of embryos during plant regeneration	Xu et al. (2011)
Callus	Hybrid PRPs (HyPRPs)	Cell elongation, enhanced size of callus	Dvorakova et al. (2012)
Whole plants	Proline transporters	Xylogenesis	Grallath et al. (2005)
Transgenic plants	Proline over-production	Increased root biomass	Kishor et al. (1995)
Transgenic plants	Proline over accumulation	Seed germination and stimulation of seedling growth	Mattioli et al. (2009b)
Transgenic plants	Proline over accumulation	Inhibition of stem growth	Nanjo et al. (1999)
Transgenic plants	Proline over accumulation	Affects flowering time	Schwacke et al. (1999), Mattioli et al. (2009b)
Transgenic plants	Proline accumulation	Flower initiation	Mattioli et al. (2008), Ghanti et al. (2011)
Transgenic plants	Proline accumulation	Male fertility	Funck et al. (2012)
Untransformed plant	Proline accumulation	More seed yield under non-stress	Bo et al. (2000)
Untransformed plant	Proline accumulation	Improves final grain production under non-stress conditions	Spoljarevic et al. (2011)
Flower buds	Proline accumulation	Bud-break	Xue et al. (2009)
Floral nectars	Proline accumulation	Attraction of pollinators	Alm et al. (1990), Carter et al. (2006), Thornburg (2007)
Whole plants	Defect in proline biosynthesis	Reduction in protein synthesis. Downregulation of cyclin genes	Wang et al. (2014)
Embryo	Defect in proline biosynthesis	Embryo lethality and defective seed development	Szekely et al. (2008), Mattioli et al. (2009b), Funck et al. (2012)
Leaf/grain/flower	Normal proline levels	Flavor compound in leaf/flower/grain	Buttery et al. (1982), Romanczyk et al. (1995), Chen et al. (2008)
Fruit	Proline accumulation	Enhances fermentability in grapevine	Stines et al. (2000)
Seed	Normal proline levels	Seed germination	Botha et al. (1992)

## Significance of Proline in Developing Seeds and in Protein Synthesis

It is known that proline plays a vital role in regulating general protein synthesis in plants. Seed mutants with opaque phenotypes have been discovered in maize (Schmidt et al., 1987; Coleman et al., 1995; Kim et al., 2006). These mutants have been found to be associated with zein (a prolamin protein in seeds) synthesis (Holding et al., 2010; Wang et al., 2011). The auxotrophic recessive opaque mutant in maize, named as *Proline responding1* (*pro1*), displays not only collapsed starchy endosperm morphology but also seedling lethality (Gavazzi et al., 1975; Ma and Nelson, 1975). Later, it was found out that mutants can be restored as normal phenotypes with the supply of exogenous proline (Racchi et al., 1978; Tonelli et al., 1984) and they have predicted that these mutants might have a defect in the biosynthesis of proline (Tonelli et al., 1986). Recently, Wang et al. (2014) reported positional cloning and functional characterization of *proline responding1* gene (*pro1*). The conclusion of Wang et al. (2014) that maize *Pro1* encodes a P5CS2 is based on the phylogenetic tree built on the P5CS full-length protein sequences taken from the genomes of *A. thaliana*, rice and maize. It has been found that this protein is localized in cytoplasm and expressed in roots, stems, silk, tassels, and kernels in these mutants and external feeding of proline rescued the viability of *pro1* seedlings. They also unraveled the mechanism related to this mutation. In these *pro1* mutants, lack of proline results in enhanced accumulation of uncharged tRNA^proAGG^. But, such an accumulation leads to a sequence of consequences, first the phosphorylation of eukaryotic initiation factor 2α (elF2α) in the mutant *pro1* and subsequently a reduction in general protein synthesis. Thus, in *pro1* mutants, lack of proline affects protein accumulation. Interestingly, Wang et al. (2014) further revealed that proline deficiency also leads to downregulation of cyclin genes at the transcriptional level and thus the cell cycle in the *pro1* mutants is significantly affected. RT-PCR analysis revealed that *CDC6* and *MCM7* genes encoding proteins involved in the formation of prereplicative complex are downregulated in *pro1* mutants, but external supply of proline induces the expression of A, B, and D cyclins, H2, H3, and H4 histones and DNA replication related genes in *pro1* mutants (Wang et al., 2014). These studies point out that lack of proline affects cyclin genes at the transcriptional level and ultimately protein accumulation in seeds.

## Proline Accumulation and Root Biomass

Though proline is produced at low levels in all tissues in unstressed conditions, it is actively transported to the roots under stress conditions. Further, it is compartimentalized into the mitochondria where it is degraded back to glutamate releasing energy in the form of FADH_2_ and NAD(P)H by two different enzymes (Szabados and Savoure, 2010). At low water potentials, maize primary roots have been shown to accumulate proline (Ober and Sharp, 1994). Maintenance of root elongation at low water potential depends on enhanced ABA content. When endogenous ABA levels were decreased either by using fluridone or *vp5* mutant, proline concentrations also decreased in the root elongation zone. But, proline concentrations in fluridone treated roots were restored back by the addition of 7 μM ABA. These experiments demonstrate that increased ABA is necessary for the deposition of proline in maize primary root growth zone. Voetberg and Sharp (1991) also demonstrated that proline levels are high toward the root apex (120 m.mol growing at a water potential of -1.6 mpa), where elongation rates of roots are maintained under a range of water deficit conditions. They suggested that proline deposition in the growing root zone is important for osmotic adjustment. But, how proline deposition plays a role in the maintenance of root elongation at low water potentials is not clearly known. Verslues and Sharp (1999) found out that proline synthesis in maize root tips from [3H]glutamate and [14C]ornithine did not increase significantly at low water potential and accounted for only a small fraction of the proline deposition. Proline is also not catabolized and utilized in the root tips under low water potential. But, increased uptake was noticed suggesting increased transport of it. Removal of endosperm from the germinated seedlings decreased the proline accumulation in root tips at low water potentials in maize (Raymond and Smirnoff, 2002). They conclude that endosperm is the source of proline accumulation in the root tips of intact seedlings constituting 10% of the free amino acids released from endosperm. Labeling experiments also revealed that proline is transported from the scutellum to other parts of the seedlings and peaked in the root tips (Raymond and Smirnoff, 2002). Though the biosynthetic capacity of roots to produce proline is low, proline accumulation perhaps is regulated by the rates of its transport and utilization. A unique salt-inducible proline transporter was isolated from barley (*Hordeum vulgare*) and found to express strongly in root cap cells under salt stress (Ueda et al., 2001). Hence, it may play a role in the root tip region when plants are exposed to salt stress. Spollen et al. (2008) analyzed the spatial distribution of transcript changes in the maize primary root elongation zone at low water potential. They concluded that different signaling and metabolic response mechanisms (wall loosening proteins in region I, and for elements of ABA and ethylene signaling) are involved in the response to water stress in different regions of the maize primary root elongation zone. Further, overexpression of *Vigna P5CS* in tobacco (Kishor et al., 1995) and *Sorghum bicolor* (Reddy et al., 2015) resulted in better root biomass under salt stress conditions. Proline accumulation by overexpression of *P5CSF129A*, a mutated version of *Vigna P5CS* in transgenic chickpea (Ghanti et al., 2011) also resulted in root elongation and higher root biomass both under salt and drought stress conditions. It appears therefore, proline may play a direct or indirect role in enhancing root biomass in transgenics under stress conditions perhaps by supplying necessary energy [FADH_2_ and NAD(P)H] and nitrogen.

Several studies related to seed germination have suggested that oxidative pentose phosphate pathway (OPPP) is important in triggering seed germination (Botha et al., 1992; Hare and Cress, 1997). Since this pathway generates NADPH, the energy may be utilized for anabolic reactions during seed germination (Besse and Buchanan, 1997). Shetty (2004) also suggested a link between proline and OPPP. But, the molecular events leading to upregulation of OPPP and its precise role in triggering seed germination is not known completely. Hare et al. (2003) have analyzed the dehydrogenase enzymes involved in OPPP during *Arabidopsis* seed germination and found activation of this pathway along with fourfold increase in proline content before the emergence of the radicle. When *AtP5CS1* gene was inserted in an antisense orientation into *Arabidopsis*, delayed emergence of radicle was noticed. Proline synthesis might replenish the NADP^+^ pool and therefore activate OPPP. This indicates a functional link between elevated proline biosynthesis and enhanced OPPP activity and coupling of both the pathways may be important in stimulating germination.

## Effects of Proline Accumulation in Reproductive Tissues under Non-Stressed Conditions

Under normal physiological (non-stressed) conditions, plants accumulate large amounts of proline during the transition to flower initiation (Chiang and Dandekar, 1995; Schwacke et al., 1999; Mattioli et al., 2009b), a common phenomenon but often an overlooked fact. Similarly, proline content in tomato flowers was 60 times higher than any other vegetative tissue and >70% of total free amino acids was noticed in pollen grains (Schwacke et al., 1999). A wide range of plants like mature fruits of citrus (Clements and Leland, 1962), pollen grains of petunia and tomato (Zhang et al., 1982; Fujita et al., 1998), ovules of broad bean (Venekamp and Koot, 1984), inflorescences and siliques of *Brassica napus* (Flasinski and Rogozinska, 1985) also contain very high levels of proline. Accumulation of proline is due to upregulation of proline biosynthetic pathway gene *P5CS*, downregulation of *ProDH* and activation of a *proline transporter T* (*ProT*) in flowers (Savoure et al., 1995; Rentsch et al., 1996; Schwacke et al., 1999; Hayashi et al., 2000). Proline accumulation predominantly in reproductive tissues suggests that it may be associated with their development (Chiang and Dandekar, 1995; Schwacke et al., 1999). It is also transported to reproductive organs as has been pointed out by Rentsch et al. (1996) and Fischer et al. (1998). These findings suggest that proline may play a role during flower initiation and its subsequent development.

Overexpression of *P5CS* in tobacco resulted in higher accumulation of proline, and enhanced flowering with more number of flowers per plant and coflorescence (cluster of flowers) formation under stress conditions (Kishor et al., 1995). Over expression of P5CS1 and over-accumulation of proline lead to early flowering and bolting promotion during the early stages of plant development in *Arabidopsis* (Mattioli et al., 2008). But, during the later stages, downregulation occurred probably because of gene silencing, leading to low proline and bushy appearance of the plants. On the contrary, antisense expression of *P5CS1* inhibited bolting in *Arabidopsis* (Nanjo et al., 1999). The *p5cs1* loss-of-function mutants exhibit delay in flowering implying that *P5CS1* plays an important role in flowering time (Mattioli et al., 2009b). They also noticed that down-regulation of *P5CS1* caused reduction in proline content, impaired flowering, and stunted growth. Funck et al. (2012) made a detailed analysis of the proline biosynthetic pathway enzymes to dissect out their roles during vegetative and reproductive development of *Arabidopsis*. In their conditions, they found alteration in flowering time in *p5cs1* mutants, but significant flowering delay in *p5cs2* mutants. It is hypothesized that the flowering delay of the 35S-P5CS1 plants as reported by Mattioli et al. (2008) could be accounted for by the homology-based co-silencing of P5CS2 triggered by the expression of P5CS1. Samach et al. (2000) found that *AtP5CS2* is a target of *CONSTANS* (*CO*), a transcriptional activator associated with flowering time. Besides, *rolD* oncogene from *Agrobacterium rhizogenes* is known to encode a functional ornithine cyclodeaminase enzyme in bacteria which ultimately converts ornithine to proline (Trovato et al., 2001). The main trait induced by overexpression of *rolD* gene in tobacco is precocity in flower setting and a strong enhancement of the flowering potential in tobacco (Mauro et al., 1996) and tomato (Bettini et al., 2003). These experiments thus point out that proline plays a vital role during flower transition, bolting, and in changing the architecture of inflorescence (coflorescence formation). However, we are not sure about the molecular events leading exactly how proline influences flowering time and changes the inflorescence architecture in higher plants when produced in optimum concentrations.

## Proline Contribution to Plant Cell Wall Architecture in Root Development and Reproductive Organs

It is a fact that proline and Hyp are the major amino acid constituents of hydrolysates of cell wall proteins. As pointed out by Kaplan and Hagemann (1991), morphogenesis takes place in plants because of differential growth of organs at the cell wall. Therefore, studies on cell walls are vital though they are highly intricate and complex. Cell wall matrix encoding proteins are enriched with proline residues which are integrated in the form of HRGPs classified into three categories: (i) moderately glycosylated EXTs; (ii) hyperglycosylated arabinogalactanproteins (AGPs); and (iii) Hyp/proline (Pro)-Rich proteins (H/PRPs).

### Role of Extensins in Cell Wall Modification During Root and Pollen Development

The extracellular matrices of plants and animals contain structural glycoproteins which are important scaffolding components. Some of these glycoproteins like collagens in animals and HRGPs of plants share functions like enhancing tensile strength (Shirsat et al., 1996) and contributing tissue integrity. In all the three subgroups, proline, and Hyp residues are abundant and play an important role in cell wall development. EXTs are self-assembling amphiphiles. They are modular in nature and contain Hyp and are usually characterized by pentameric Ser-Hyp peptide repeats. EXTs exhibit 35–65% glycosylation on Ser-(Hyp)_n__≥__2_ motifs and the side chains contain oligosaccharides of arabinose and galactose (Showalter, 1993; Kieliszewski and Lamport, 1994). The genome of *A. thaliana* encodes 63 putative EXTs with high amino acid sequence similarity between them. Out of the 63 members, some are classical, while others are EXT-like chimeras and hybrid EXTs (Lamport et al., 2011). It is not clear whether they have redundant or specific functions to carry in different cell types of various plant tissues. EXTs exhibit repetitive nature of their sequences with many post-transcriptional modifications in plant genomes like signal peptide processing in the ER, hydroxylation of proline to Hyp residues by P4H, *O*-glycosylation on Hyp and Ser residues with chains of up to four linear Ara residues on each Hyp by arabinosyltransferases (Velasquez et al., 2011; Ogawa-Ohnishi et al., 2013), and cross-linking of glycosylated EXTs at the tyrosine residues in the cell wall (Jackson et al., 2001; Price et al., 2003; Lamport et al., 2011; Hijazi et al., 2014) forming intra- and inter- EXT linkages (Nunez et al., 2009; Dick-Perez et al., 2011). This helps EXTs to form a three-dimensional structure of protein and to interact with pectins in the cell walls (Dick-Perez et al., 2011). The coordination of EXTs with other components and their functions during cell wall architecture and plant development are not completely known. This is mostly because of their modular nature, existence in large numbers, hydroxylation by different P4Hs, complex structure, and redundant expression of these proteins in the same plant tissues (Genevestigator database, http://www.genevestigator.com). EXTs can also interact with pectins and serve as templates for cell wall deposition (Cannon et al., 2008; Lamport et al., 2011). Recently, Tan et al. (2013) found an ARABINOXYLAN PECTIN ARABINOGALACTAN PROTEIN1 (APAP1) in *A. thaliana* which consists of pectin and arabinoxylan that are covalently linked to an AGP. They suggested a role for APAP1 proteoglycan in plant cell wall architecture and function. However, the exact coordinated control of EXTs and pectin interaction during cell wall formation is unclear.

Several groups have used root hair as a model to study the functions of EXTs (Park et al., 2011; Velasquez et al., 2011). Mutants lacking the synthesis of wall polymer in the root hair are impaired in growth (Diet et al., 2006; Ringli, 2010; Park et al., 2011; Velasquez et al., 2012). Since posttranslational modifications of HRGPs are carried out by (P4Hs), this defines the subsequent *O*-glycosylation sites in EXTs (mostly arabinosylated) and polarized growth in root hairs. *O*-glycans enhance the HRGP solubility and resistance to proteolytic degradation (Kieliszewski et al., 1989; Shpak et al., 2001; Lamport et al., 2011). Velasquez et al. (2011) proved that *O*-glycosylation on EXTs is essential for cell-wall assembly and root hair elongation. Root hairs develop by tip growth akin to pollen tubes, axons, and fungal hyphae. Velasquez et al. (2015) recently carried out genetic analysis related to the regulation of P4H. They proved that P4H5 and P4H2 and P4H13 are pivotal for root hair tip growth. The impact of deficient proline hydroxylation on the cell wall architecture has also been shown by them. Their results demonstrate that peptidyl-proline hydroxylation on EXTs is required for cell wall assembly and therefore root hair elongation in *Arabidopsis*. The above works point out that changes in the *O*-glycosylation status impact the functions of EXTs during cell wall assembly, cell shape, and expansion. Genes that encode *P4Hs* are also linked to hypoxia, and P4H proteins are regarded as oxygen sensors under hypoxic stress. Zou et al. (2011) found out that *P4H* genes are subjected to alternative splicing in roots of maize seedlings under waterlogging conditions. The diverse transcripts generated due to alternative splicing are expressed at different levels clearly indicate that *ZmP4H* genes are under specific control by post-transcriptional regulation under waterlogging stress in maize. Hall and Cannon (2002) identified that cell wall Hyp-rich glycoprotein *ROOT-SHOOT-HYPOCOTYL-DEFECTIVE* (*RSH*) is necessary for normal embryo development in *Arabidopsis*. Xu et al. (2011) further found out the role of HRGPs during somatic embryogenesis of *Musa* sp. Their results suggest that HRGPs play a vital role in regeneration and germination of embryos during early plant development *via* somatic embryogenesis (**Table 1**). Proper localization and appropriate quantities of HRGPs seem to be critical for the formation and regeneration of somatic embryos. But, the exact mechanism by which HRGPs trigger somatic embryogenesis in callus or suspension cultures is not known. Thus, the functions proline-rich and Hyp-rich glycoproteins appear to be complex, but clearly associated with developmental regulation in different plant systems. Using protein blots and immunohistochemistry, EXTs have been found to be abundantly expressed *in vivo* for pollen tubes and transmitting tissues (Zhang et al., 2014). When inhibitors of HRGP were used, decreased pollen tube growth and stylar length were recorded. It appears that HRGPs especially EXTs play a critical role in the pollen tube and style cell growth. Wu et al. (2001) demonstrated that EXTs are involved in pollen recognition and fertilization. These evidences along with the works of Cannon et al. (2008), Ringli (2010), Lamport et al. (2011), and Velasquez et al. (2012) infer that cross-linked EXTs play pivotal roles during cell expansion and growth of pollen tube.

### Arabinogalactan Protein Involvement in Vegetative and Reproductive Growth

Arabinogalactan proteins are Hyp-rich extracellular proteins with a high proportion of sugars (up to 90%). Among them, arabinose and galactose are predominant residues, but minor sugars like rhamnose, fucose, glucuronic, acid, and xylose are common (Nothnagel, 1997; Showalter, 2001). AGPs are characterized by extensive glycosylation with arabinose, galactose or both and analogous to animal proteoglycans (Clarke et al., 1979; Lord and Sanders, 1992). AGPs possess repetitive Alanine (Ala) or Serine (Ser) motifs that interact with Hyp molecules. They are chemically stable, and resistant to proteolytic enzymes in their native state due to the presence of carbohydrates (Fincher et al., 1983). AGPs contain glycosylphosphatidylinositols (GPIs) which tethers them to the plasma membranes in plants (Schultz et al., 2004). The biosynthesis, structure, expressions, and functions of AGPs have been well studied and reviewed (Showalter, 2001; Ellis et al., 2010; Tan et al., 2012; Lamport and Várnai, 2013; Nguema-Ona et al., 2013; Knoch et al., 2014). Cassab (1998) pointed out that carbohydrate groups (D-galactose and L-arabinose) are mostly linked by *O*-glycosylation to the hydroxyl group of Ser and Hyp of the AGP backbone. However, diversity exists in the degree and pattern of *O*-glycosylation depending on the type and length of glycan chains attached to the proteins. Many studies suggest that AGPs play important roles in vegetative and reproductive growth. AGPs were observed throughout the plant kingdom, in leaves, stems, roots, floral parts, and seeds (Fincher et al., 1983; Nothnagel, 1997). They were found in plasma membranes, cell walls, intracellular multivesicular bodies, and also as secretions to intercellular spaces (Nothnagel, 1997).

Some AGPs are also implicated with redundant architectural functions of cell walls (Bosch et al., 2001). Several lines of evidence indicate that AGPs are associated with growth and differentiation in lower as well as in higher plants. For example, when inhibitors of AGP biosynthesis were used, the growth of leaf primordia was suppressed in a liverwort (Basile and Basile, 1993). Yariv reagent inhibited cell division in cell suspension cultures and roots in carrot and *Arabidopsis*, respectively, (Willats and Knox, 1996; Ding and Zhu, 1997). This indicated that AGPs are involved in cellular growth and development. While certain AGPs promote somatic embryogenesis in *Daucus carota*, (Kreuger and van Holst, 1993), and *Picea abies* (Egertsdotter and von Arnold, 1995), others inhibit it as noticed by Toonen et al. (1997) in carrot.

AGPs were noticed in xylem, stylar transmitting tissues and cell suspension cultures. In targeting certain AGPs to the specific cell surface locations, the role of certain sequence determinants in the carbohydrate or protein need to be determined. Their multi-location (organs, tissues, and cell-types) occurrence indicates specific functions of AGPs in different tissues like flowers and other cell types (Pennell and Roberts, 1990). Many AGPs possess a C-terminal *Ole e* 1 domain (pollen allergens, named after a secreted protein isolated from *Olea europeae* pollen; Villalba et al., 1994). Developmental and tissue specific expressions were noticed for several AGPs in different plants (Pennell et al., 1991; Chen et al., 1994; Gao and Showalter, 2000), consistent with the assumption that they play a role in plant development. Altered expression of AGP in pea plants has changed the sexual development as shown by Pennell and Roberts (1990). Some AGPs like stylar transmitting tissue-specific PRPs (TTS proteins) play vital roles in reproductive growth and development like pollen-pistil interactions (Bosch et al., 2001; Hancock et al., 2005). When the gene knockdown experiments were carried out, TTS proteins have been found important for pollen–pistil interactions. This may be possible for TTS proteins which act as cell surface adhesives (Wu et al., 2000). AGPs are also implicated in cellular signaling events (Schultz et al., 1998). They are the important components in plant exudates and gums, as shown in the case of gum arabic extracted from *Acacia senegal* (Serpe and Nothnagel, 1999) and used as an additive in the food industry. Type II arabinogalactans have also been found to stimulate animal immune systems and thus may be useful in medicine as well (Yamada and Kiyohara, 1999). Further, chimeric proteins containing AGP domains may interact with polysaccharides. In this connection, the experiments of Griffiths et al. (2014) have proved that *CELLULOSE SYNTHASE5* (*CESA5*) and the *SALT-OVERLY SENSITIVE5* (*SOS5*), a Fasciclin-AGP are required for mucilage adherence in seeds of *A. thaliana*. They conclude that *SOS5* mediates adherence through pectin (Griffiths et al., 2014). AGPs are involved in cell–cell/intercellular communications involved in tracheary element differentiation (Motose et al., 2001) and thus AGPs are found to be associated with the differentiation of xylem and programmed cell death (Schindler et al., 1995; Gao and Showalter, 2000). Thus, AGPs perform several functions in different aspects of plant growth and development, though the underlying molecular mechanisms are not clearly known.

### Pursuit of Hyp/Pro-Rich Proteins (H/PRPs) in Plant Development

Hyp/Pro-rich proteins belong to the HRGP superfamily like that of EXTs and AGPs. Based on repetitive motifs and domain organization, PRP proteins are classified into three subtypes; (a) repetitive PRPs, (b) non-repetitive PRPs, and (c) multi-domain hybrid PRPs (Jose-Estanyol and Puigdomenech, 2000). Proline residues in PRPs are hydroxylated and form Hyps which are then glycosylated as mentioned earlier. But, *O*-glycosylation of H/PRPs and their interactions with polysaccharides is not completely known. Twelve H/PRP proteins have been identified so far in *Arabidopsis thaliana*, carrot, cotton, tobacco, common bean, capsicum, and petunia (Hijazi et al., 2014). Bradley et al. (1992), and Frueauf et al. (2000) pointed out that H/PRPs may be cross-linked in cell walls, but evidence is lacking for such an assumption.

Pro-rich proteins are involved in cell elongation (Ito et al., 1998), abscission and senescence (Merkouropoulos and Shirsat, 2003). As shown in **Table 1**, HRGPs are essential for the structure and function of cell wall as well as plasma membrane-cytoskeleton continuum (Showalter, 1993; Showalter et al., 2010). It is highly interesting to note that the PRP genes show developmental stage-, organ-, tissue-, and even cell-specific expressions (Hong et al., 1989; Ye and Varner, 1991; Wyatt et al., 1992). Hong et al. (1987, 1989) reported the characterization of three developmentally regulated proline-rich cell wall protein genes from *Glycine max*. Marked differences were observed in the pattern of expression of PRPs in different organs like hypocotyls, roots, stems (Wyatt et al., 1992), immature embryos (Jose-Estanyol et al., 1992), immature seed coats and developing seeds and in cells that are undergoing lignifications (Vignols et al., 1999). Fowler et al. (1999) characterized and expressed four proline-rich cell wall protein genes in *Arabidopsis* encoding two distinct subsets of multiple domain proteins. Their studies support a model for the involvement of PRPs in specifying cell-type specific wall structures, and provide the basis for a genetic approach to dissect the function of PRPs during growth and development. While *S. bicolor PRP1* (*SbPRP1*) mRNA is abundant in elongating regions of hypocotyl epidermal cells, *SbPRP2* mRNA is more expressed in phloem cells. Further, it has been found that *SbPRP3* mRNA is localized in the endodermoid layer of cells in the hypocotyl elongating region (Wyatt et al., 1992). Bernhardt and Tierney (2000) demonstrated that expression of *AtPRP3*, a proline-rich structural cell wall protein is regulated by cell-type-specific developmental pathways involved in root hair (single cells that develop by tip growth and are specialized in absorption of nutrients) formation. They noticed enhanced expression of *AtPRP3*/β-glucuronidase (GUS) in roots of transgenic seedlings treated with 1-aminocyclopropane-1-carboxylic acid (ACC) or α-naphthaleneacetic acid, two compounds known to promote root hair formation. The results indicate that *AtPRP3* is regulated by developmental pathways involved in root hair formation (Bernhardt and Tierney, 2000). Overexpression of *GhPRP5* in *A. thaliana* resulted in smaller cell size, but knock-down of *GhPRP5* expression by RNA interference in cotton increased the fiber length (Xu et al., 2013). Their data suggest that this protein participates in modulating fiber development of cotton.

## Involvement of PRPs as Signaling Proteins in Reproductive Tissues

Pro-rich proteins perform a whole spectrum of functions like providing structural integrity to mediate cell–cell integrations and communications (Wu et al., 2001). They are also involved and play a vital role in wall assembly and remodeling during cell growth and development (Ellis et al., 2010; Doblin et al., 2010). Gibbon et al. (1998) characterized several profilin isoforms from maize pollen and showed that their function depends on interaction with proline-rich motifs. They found out that profilin isoforms expressed in a single cell can have different effects on actin in living cells and the poly-L-proline binding function of profilin may have important consequences for the regulation of actin cytoskeletal dynamics. Two stylar transmitting tissue specific PRPs (TTS-1 and TTS-2) have also been reported in tobacco (Cheung et al., 1993). *TTS-1* and *TTS-2* mRNAs have been induced in floral buds, during the later stages of flower development especially when style elongation is rapid and are active at anthesis (Cheung et al., 1993). The occurrence of PRPs in transmitting tissues has been predicted to play a vital role in maintaining the structural integrity of the style. Transgenic *Arabidopsis* overexpressing *Atext1* displayed altered inflorescence and affected stem thickening and height (Roberts and Shirsat, 2006). However, the functions of all PRPs are not completely known during different developmental stages, thus leaving a gap in our understanding of PRPs.

## Role of PRPs in Cell Wall Modification under Stress

Genes that encode PRPs and the localization of these proteins in plant cell walls have been studied (Cassab and Varner, 1988; Showalter and Varner, 1989; Cassab, 1998). During growth, differentiation and also during different environmental stress conditions, cells may expand up to 200 times their original length. Water stress could result in alterations in cell volume and shape, as well need to deal with loss of turgor (Wakabayashi et al., 1997). Therefore, cell walls must possess tensile strength to withstand the turgor pressures. This involves very large chemical and biochemical modifications of cell wall constituents including cell wall proteins representing 1 to 2% of the genome in plants such as *Arabidopsis* (Somerville et al., 2004). Cell wall protein transcripts are also affected under salt, drought, and temperature stress conditions (Covarrubias et al., 1995). PRPs are insolubilized in the cell walls with the involvement of H_2_O_2_-mediated oxidative cross linking and it precedes the expression of transcription-dependent defences. Though cell wall bound PRPs may play a role in the structural integrity of plant cells, participate in defence related activities, and plant cell surface interactions as pointed out by Roberts (1989), Varner and Lin (1989), interestingly, the mRNA levels of PRPs increase in response to water scarcity in higher plants (Creelman and Mullet, 1991; Colmenero-Flores et al., 1997).

Battaglia et al. (2007) characterized two proline-rich glycoproteins of 33 and 36 kDa (p33 and p36) which are found in the cell wall soluble fraction of common bean (*Phaseolus vulgaris*). They observed the highest accumulation of these proteins in growing regions, predominantly in phloem tissues in response to drought indicating that cell wall modifications are induced in actively growing cells of common bean. Gothandam et al. (2010) reported a flower specific PRP from rice (*OsPRP3*) that is expressed during the late stages of flower development. While *AtPRP3* is associated with root hair formation, overexpression of *Oryza sativa PRP3* (*OsPRP3*) showed an increase in cold tolerance compared to the wild-type plants. They showed that this *OsPRP3* enhances cell wall integrity in the cold tolerant plants. Knockout mutants displayed defects in floral organogenesis suggesting a role for PRPs in reproductive tissues. Tseng et al. (2013) reported ABA-induced expression of a family of four genes, *REPETETIVE PROLINE-RICH PROTEIN* (*RePRP*) in rice. These genes encode proline-rich glycoproteins with highly repetitive PX_1_PX_2_ motifs, RePRP1, and RePRP2. Their work also revealed that ABA treatment increases *RePRP* expression and that these proteins are localized to the plasma membrane. It has been found that knockdown of the expression of *RePRP1* and *RePRP2* decreases the sensitivity of roots to ABA. Thus, these experiments reveal that RePRP proteins play an essential role in ABA/stress regulation of root growth and development. It has also been noticed that rice RePRPs interact with arabinogalactan polysaccharide in a dosage-dependent manner (Tseng et al., 2013). Zhan et al. (2012) recently identified a PRP called *SICKLE* (*SIC*) in *Arabidopsis*. This protein is critical for not only development in *Arabidopsis* but also in imparting abiotic stress tolerance. The loss-of-function *sic-1* mutants displayed reduction in plant height, delay in flowering, and abnormal inflorescence phyllotaxy. These mutants accumulated reduced levels of a subset of miRNAs and transacting siRNAs but enhanced levels of primary miRNAs than the controls. The *sic-1* mutant plants are also sensitive to cold and salt stresses (Zhan et al., 2012). Therefore, it appears that SIC is a PRP associated with the biogenesis of some miRNAs and degradation of some spliced introns and is vital for plant development.

## Hybrid Proline-Rich Proteins (HyPRPs) are Crucial Players in Cell Elongation

Out of the three subclasses of PRPs, one of them contains several copies of POVEKPOVXK motif (Hong et al., 1990), while the other two subclasses show a hybrid structure and are called hybrid proline-rich proteins (HyPRPs). Plant HyPRPs represent putative cell wall proteins consisting of a repetitive proline-rich N-terminal domains and a conserved C-terminal domain. They are unique to seed plants and analysis of the families from different plant species suggests rapid diversification of their sequences and expression patterns (Dvorakova et al., 2007). Their functions are not clearly known, but their occurrence indicates that they may be involved in basic cellular processes (Dvorakova et al., 2012). To understand the functions of HyPRPs, Dvorakova et al. (2012) modulated the expression of three *HyPRP* genes in tobacco and potato. Transgenic plants displayed cell elongation, and enhanced size of calli. HyPRPs contain two regions, and one of them is rich in proline and the other in cystein residues (Deutch and Winicov, 1995). Further, it has been shown that the third sub-class, namely NHyPRPs displays high percentage of proline residues organized in repetitive sequence motifs in C-terminus region (Castonguay et al., 1994; Menke et al., 2000). Thus, it appears that HyPRPs are important players in plant cell elongation, though the molecular mechanism is not yet elucidated.

## Proline-Rich Extensin-Like Receptor Kinases (PERKs)

A family of receptor kinase-like proteins has been discovered that are involved in cell wall signaling. In *A. thaliana*, 11 members of the PERK family have been discovered which share some features of EXTs. Altered expression of PERK receptor kinases resulted in changes of plant growth and floral organ formation (Haffani et al., 2006). In a novel work, it has been elucidated by Bai et al. (2009) that the gene *PERK4* (coding for a protein kinase located in the plasma membrane) is required for the ABA-dependent influx of Ca^2+^ and normal ABA sensitivity in seeds and roots of *A thaliana*. Their experiments suggest that PERK4 is associated in ABA perception and also might interact with wall polymers. Thus, PRPs are implicated in cell wall signal transduction cascades. Further work is necessary since it is not yet clear about how many PERK proteins are associated with this process and also their additional roles if any during growth and stress tolerance.

## Proline Deficiency Affects the Biosynthesis of Cell Wall Matrix Proteins

The experiments conducted by Cooper et al. (1994) demonstrated initially in tobacco that HRGPs are critical for cell morphology and osmotic stability. Strong evidence has come later from Nanjo et al. (1999) who created *Arabidopsis* plants with antisense *P5CS*. Interestingly, the transgenics displayed morphological defects in leaves and elongation of inflorescences. Further, they observed that protein biosynthesis was normal in proline deficient plants, but structural proteins localized in the cell walls were strikingly altered (Nanjo et al., 1999) indicating a direct evidence for a key role of proline in cell walls. Specifically, proline and Hyp contents were reduced in the cell wall preparations of *Arabidopsis* transgenic leaves. They pointed out that PRPs and HRGPs are responsible for morphological abnormalities observed in the leaves of antisense *Arabidopsis* plants since these proteins provide mechanical support for cells under stressed conditions. Such a role in cell morphology and the mechanical support for the PRPs and HRGPs also has been demonstrated by Showalter (1993) and Munoz et al. (1998). Thus, proline-related growth abnormalities are correlated well with the effects of proline on plant cell wall development. Wang et al. (2014) found out that proline deficiency causes downregulation of cyclin genes and affects general protein synthesis. However, they did not find out its effect on the cell wall proteins. It would be interesting to find out if there would be any changes that take place in H/PRPs associated with cell wall development. Several overexpressions and knockout mutations of plants containing PRPs and HRGPs are needed to demonstrate the exact role(s) of these proteins during cell wall synthesis and growth.

## Proline Transporters Play an Important Role in Plant Development Including Xylogenesis

Proline transporters have been isolated from several species and three from *Arabidopsis* (*AtProTs*) so far (Rentsch et al., 1996; Schwacke et al., 1999; Fujiwara et al., 2010; Lehmann et al., 2011). Transcript levels of *Arabidopsis ProT2*, mangrove *ProT* homologues, barley *HvProT* as well as proline levels are elevated under salt stress (Rentsch et al., 1996; Hibino et al., 2001; Ueda et al., 2001; Waditee et al., 2002). Like in bacteria, proline transporters also act as glycinebetaine transporters in plants but with different affinities. Further, they also transport other compatible solutes like γ-aminobutyric acid (GABA) in plants (Grallath et al., 2005; Fujiwara et al., 2010). Though intracellular localization, substrate selectivity, and affinity of the three AtProTs are similar, they exhibited differential expression patterns in various plant organs indicating differential transport of these molecules. By fusing transporter genes with GUS, they demonstrated that *AtProT1*, *AtProT2*, and *AtProT3* are localized in lignified tissues like phloem of all organs, epidermis, and cortex of roots and in epidermis of leaves respectively, (Grallath et al., 2005). *ProT1* isolated from tomato has shown to be expressed specifically in pollen grains (Schwacke et al., 1999). Further, Lehmann et al. (2010) clearly showed a relation between uneven distribution of proline in the lower epidermis and rest of the leaf with the expression levels of *AtProT3*. In spite of the fact, that single, double, and triple knockout mutants of the *AtProT1*, *AtProT2*, and *AtProT3* genes responsible for proline transport in plants have been isolated and characterized, these mutants did not reveal any differences when compared to wild type plants (Lehmann et al., 2011). This underlays the complex situation and therefore needs further elucidation. The functions of these transporters might be highly redundant and possibly complemented by other transporters, since transporters mediating proline uptake across the plasma membrane have also been noticed in the amino acid transporter (ATF) or amino acid/auxin permease (AAAP) and in the amino acid-polyamine-choline (APC) families (Rentsch et al., 2007). Along with glutamate, amino acid permease (AAP) family mediates proton coupled uptake of neutral amino acids such as proline (Frommer et al., 1993; Lee and Pallas, 2007; Schmidt et al., 2007). Couturier et al. (2010) isolated *AAP11* from *Populus trichocarpa* and overexpressed it in poplar and yeast. *PtAAP11* was highly expressed in differentiating xylem cells in different organs. Further, functional characterization revealed that it is a high affinity amino ATF, more particularly for proline. Therefore, the authors suggested that *PtAAP11* may play an important role in xylogenesis by providing proline which is required for xylem cell wall proteins in poplar. Lysine-histidine transporter (LHT) family transports neutral and acidic amino acids including proline (Lee and Tegeder, 2004). Contrary to the AAP and LHT families, ProTs transport proline and other compatible solutes like GABA but not other proteogenic amino acids (Rentsch et al., 1996). The fact that both low and high affinity-proline transporters exist in plants implicate that they play a role in general transfer of nitrogen and also proline for its specific functions in different cells and tissues. Selective transport of proline by ProTs and also their expression analysis during drought/salt stresses or in pollen grains suggests that they play a vital role in proline homeostasis both under stress and non-stress conditions (Rentsch et al., 1996; Ueda et al., 2001; Waditee et al., 2002; Lehmann et al., 2010). The list of proline/betaine transporters and transport mutants known from different plants is shown in **Table 2**. In summary, proline transporters (AAPs) play an important role in plant development by providing proline as a source of nitrogen and energy which is required for lignifications, xylem differentiation and cell wall modification during plant development (as a component of plant cell wall proteins). But the exact mechanisms how proline can mediate these functions are not known.

**Table 2:** List of proline/betaine transporters and transport mutants known from different plants.

Name of plant	Type of transporter/mutant	Affinity for proline	Tissue specific expression/function	Reference
*Amaranthus tricolor*	*AmtBet/ProT1*	Low compared to betaine	Not known	Yamada et al. (2011)
*Arabidopsis thaliana*	*AtProT1*	High	Phloem parenchyma in all organs, pollen grains	Rentsch et al. (1996), Grallath et al. (2005)
*A.thaliana*	*AtProT2*	High	Root cortex, epidermis, seedling after salt and drought stress	Rentsch et al. (1996), Grallath et al. (2005)
*A.thaliana*	*AtProT3*	High	Leaf epidermis, sepal	Rentsch et al. (1996), Grallath et al. (2005)
*Avicennia marina*	*AmBet/AmProT1*	High	Leaf and root after salt stress	Waditee et al. (2002)
*A. marina*	*AmBet/AmProT2*	High	Leaf and root after salt stress	Waditee et al. (2002)
*A. marina*	*AmProT3*	Not known		Yamada et al. (2011)
*A. thaliana*	*raz1 mutant*	-	Affected in the high affinity uptake of proline	Verbruggen et al. (1993)
*Atriplex gmelinii*	*AgBet/ProT1*	Low compared to betaine	Not known	Yamada et al. (2011)
*A. hortensis*	*AhProT1*	High	Roots	Shen et al. (2002)
*Beta vulgaris*	*BvBet/ProT1*	High	Xylem and phloem parenchyma cells, petiole, leaf	Yamada et al. (2009, 2011)
*Chlorella vulgaris*	*pup mutant*	–	Impaired in alanine, glycine, proline, and serine transport	Sauer and Tanner (1985)
*Elaeis guineensis*	*EgProT1*	High	Not known (may be uptake into roots)	Yamada et al. (2011)
*Hordeum vulgare*	*HvProT1*	Not known	Root cap, cortex, stele, phloem after salt stress	Ueda et al. (2001, 2008), Fujiwara et al. (2010)
*H. vulgare*	*HvProT2*	High	Leaves, root, plasma membrane, mestome sheaths, lateral root caps	Ueda et al. (2001), Fujiwara et al. (2010)
*Lycopersicon esculentum*	*LeProT1*	High	Flowers, mature and germinating pollen	Schwacke et al. (1999)
*L. esculentum*	*LeProT2*	Not known	Could not be detected with RNA gel blot	Schwacke et al. (1999)
*L. esculentum*	*LeProT3*	Not known	Could not be detected using RNA gel blot	Schwacke et al. (1999)
*Oryza sativa*	*OsProT1*	Not known	All organs	Igarashi et al. (2000)
*A. thaliana*	*AtAAP1*	Low in yeast High in *Xenopus*	Expression in the seed, root epidermis, root tip, root hairs	Frommer et al. (1993)
*A. thaliana*	*AtAAP2*	High	Vascular bundles in leaf, root, silique, flower	Kwart et al. (1993), Fischer et al. (1998), Hirner et al. (1998)
*A. thaliana*	*AtAAP3*	High	Root, tip of filament and seedlings (cotyledons)	Fischer et al. (1995), Okumoto et al. (2004)
*A. thaliana*	*AtAAP4*	High	Stem, source leaf, sink leaf, flower	Fischer et al. (1998)
*A. thaliana*	*AtAAP5*	High	Source leaf, stem, flower, fruit, root	Fischer et al. (1998)
*A. thaliana*	*AtAAP6*	Low	Xylem parenchyma in aerial parts, sink leaf, root	Rentsch et al. (1996), Okumoto et al. (2002)
*O. sativa*	*OsAAPs*	Not known	Not known	Tegeder and Ward (2012)
*Solanum tuberosum*	*StAAP1*	Not known	Source leaf	Fischer et al. (1998)
*S. tuberosum*	*StAAP2*	Not known	Stem	Fischer et al. (1998)
*Ricinus communis*	*RcAAP1*	Not known	Sink leaf, source leaf, root and seedling	Fischer et al. (1998)
*R. communis*	*RcAAP2*	Not known	Sink leaf, source leaf, root, and seedling	Fischer et al. (1998)
*A. thaliana*	*AtLHT1*	Low	Leaf epidermis and mesophyll, root tip, stem, petals, sepals	Kwart et al. (1993), Chen and Bush (1997)
*A. thaliana*	*AtLHT2*	Low	Tapetum, pollen	Lee and Tegeder (2004), Foster et al. (2008)
*O. sativa*	*OsLHTs*	Not known	Not known	Tegeder and Ward (2012)
*A. thaliana*	*AtCAT1*	High	Flower, veins of root, leaf, silique, stem	Frommer et al. (1995)
*A. thaliana*	*BASIC AMINO ACID CARRIER2*	Not known	Mitochondria Mutations in *BAAC* contribute to proline accumulation in response to hyperosmotic stress	Toka et al. (2010)

## Mutations in *p5cs2* and *p5cr* Cause Embryo Lethality and Affect Seed Development

The amount of free proline and its metabolism modulates transition to flowering, pollen, and embryo development. Several studies found that immature/mature seeds of *Vicia faba* and *Arabidopsis* accumulate proline before ripening (Venekamp and Koot, 1984; Chiang and Dandekar, 1995; Schmidt et al., 2007). In support of it, concomitant expression of genes encoding enzymes of proline metabolism was observed in seeds (Armengaud et al., 2004; Deuschle et al., 2004; Hur et al., 2004; Szekely et al., 2008). It was noticed that *P5CS2* is expressed mostly in actively dividing meristematic cells, developing tissues, callus, and cell suspension cultures. *p5cs2* mutations cause embryo abortion during late stages of seed development though both *P5CS* mRNAs are detectable throughout embryonic development under warmer climate (Szekely et al., 2008). Their experiments indicated exogenous proline can rescue *p5cs2* mutants by *ex vivo* cultivation of developing seeds, but the mutant plants undergo aberrant development and become sterile. It has also been reported that homozygous *p5cs2* mutant plants died before the onset of flowering. Therefore, specific role of *P5CS2* in reproductive development could not be analyzed (Szekely et al., 2008; Mattioli et al., 2009b). When *Arabidopsis* plants were simultaneously silenced or co-suppressed with *P5CS1* and *P5CS2* genes, they resulted in retarded growth, delayed flowering and reduced apical dominance (Nanjo et al., 1999; Mattioli et al., 2008). The above results indicate that *Arabidopsis P5CS1* is insufficient for compensation of developmental defects caused by knockout of *P5CS2*. Expression of *P5CS2-GFP* was observed in leaf primordia, where the levels of *P5CS1-GFP* levels are very low. *P5CS2-GFP* also displayed cell-type specific subcellular localization pattern compared to *P5CS1-GFP* in root tips, leaves, flower organs, and embryonic cells (Szekely et al., 2008). Taken together, the data demonstrate that these two genes have non-redundant functions in plants. In *A. thaliana*, Funck et al. (2012) did not observe developmental defects in single mutants devoid of *P5CS1* gene. *p5cs1*/*p5cs2* double mutants displayed pollen sterility, while egg cells are fertile. Thus, these results emphasize the role(s) of P5CS in pollen fertility. *p5cs2* T-DNA insertion lines have been described as embryo lethal or conditionally embryo lethal. It has been found that P5CS2 is not essential for sexual reproduction (Funck et al., 2012). They obtained homozygous *p5cs2* mutants that are viable and could produce fertile seeds by *in vitro* culture of immature mutant seeds on MS medium supplemented with 60 mM sucrose and 2 mM proline. This allowed generating homozygous plants that are phenotypically normal, but showed reduced growth compared to the wild type plants. Under short day and low-light conditions, these plants produced viable seeds. Funck et al. (2012) pointed out that embryo lethality in heterozygous *atp5cs2* plants might be due to premature desiccation of homozygous embryos that develop more slowly than heterozygous embryos. In a contrast to *p5cs1*/*p5cs2* double mutants, normal pollen fertility was observed in the absence of a functional *P5CR* gene indicating that *P5CR* is not needed for pollen fertility. These results implicate that *P5CS1/P5CS2* genes are crucial for pollen fertility, but not *P5CR*. It has been found that *p5cr* mutations cause embryonic lethality, however. Disruption of *P5CR* gene using T-DNA insertions resulted in embryo defective mutation *emb2722* (Meinke et al., 2008) in *Arabidopsis*. They reported arrest of the embryo development after the second division of the embryo proper in *p5cr* mutants. All attempts to rescue putative homozygous *p5cr* mutant embryos *in vitro* by proline feeding failed. Their efforts to promote embryo development of homozygous seeds in siliques of heterozygous parents *in situ* by feeding the proline exogenously or induction of internal proline accumulation through salt stress were not fruitful. Thus, activities of both P5CS and P5CR enzymes have been found essential for sexual reproduction in plants.

## Conclusion

Proline not only participates in protein synthesis, but regulates several important functions like osmotic adjustment and protection of proteins during stress conditions. Its multifarious functions are always enigmatic in plants. However, it is increasingly becoming evident that proline modulates a wide-array of functions like regulation of cyclin genes at the transcriptional level, cell wall elongation and modifications, xylogenesis, stem elongation, root, and shoot growth, inflorescence architecture, embryo formation/seed development, and seed germination during the life cycle of a plant. Deficiency in proline biosynthesis leads to abnormal plants and cell wall defects, thus implicating its role in structural proteins. But, clear evidence is lacking for attributing several specific functions to this amino acid. We do not know yet how the genes like *P5CS1*, *P5CS2*, *P5CR*, *OAT, PDH*, and *P5CDH* interact with other genes and we still do not have an interactome map of the network of genes. Unless such maps are available along with their experimental validation, we cannot pinpoint the exact metabolic functions of proline other than its role in primary metabolism in protein biosynthesis.

## Author Contributions

All the authors of the manuscript meet the essential criteria of the publication. All authors have read and approved the manuscript.

## Conflict of Interest Statement

The authors declare that the research was conducted in the absence of any commercial or financial relationships that could be construed as a potential conflict of interest.
